# Resonance assignments of the human receptor interacting protein kinase 1 (RIPK1) in its fibrillar conformation

**DOI:** 10.1007/s12104-025-10249-y

**Published:** 2025-09-15

**Authors:** Paula Polonio, Miguel Mompeán

**Affiliations:** 1 Instituto de Química Física “Blas Cabrera” - Consejo Superior de Investigaciones Científicas (IQF-CSIC), Serrano 119, Madrid, 28006 Spain; 2 Universidad Autónoma de Madrid, Escuela de Doctorado, Francisco Tomás y Valiente 2, Madrid, 28049 Spain

**Keywords:** Amyloid, RHIM, Cryogenic CPMAS probes, Solid-state NMR, RIPK1, Necroptosis

## Abstract

Receptor-interacting protein kinase 1 (RIPK1) is a key regulator of necroptotic signalling that forms functional amyloid fibrils through its RIP Homotypic Interaction Motif (RHIM). Here, we report the solid-state NMR chemical shift assignments for the rigid amyloid core of human RIPK1 fibrils, encompassing residues 529–552 within the RHIM. Assignments were obtained from uniformly ^13^C,^15^N-labeled protein diluted with unlabeled protein and measured using cross-polarization magic angle spinning (CPMAS) experiments on a cryogenic probe. The dataset includes backbone and side-chain resonances for the ordered region and provides a basis for high-resolution structural and dynamics studies of RIPK1 and related RHIM-containing assemblies.

## Biological context

Receptor-interacting protein kinase 1 (RIPK1) is a central signaling hub that regulates diverse cellular outcomes, including inflammation, apoptosis, and necroptosis. Its role in coordinating life-or-death decisions in the cell depends on a combination of catalytic activity, protein-protein interactions, and the ability to form supramolecular assemblies (Christofferson et al. 2014; Newton 2020; Clucas and Meier 2023). Understanding the structural basis of these interactions is critical for dissecting how RIPK1 switches between different signaling programs (Chan et al. 2015; Yao et al. 2025).

The modular architecture of human RIPK1 includes three distinct regions, namely: (1) an N-terminal kinase domain that undergoes multiple post-translational modifications (PTMs) (Cho et al. 2009; Delanghe et al. 2020; Clucas and Meier 2023); (2) a C-terminal death domain (DD) that mediates interactions with other DD-containing receptors and adaptors (Meng et al. 2018); and (3) an intrinsically disordered intermediate region that contains a conserved tetrapeptide motif, I/V-Q-I/V/L-G, known as the RIP Homotypic Interaction Motif (RHIM). The RHIM enables RIPK1 to engage in homotypic and heterotypic interactions with other RHIM-containing proteins and to assemble into amyloid fibrils (Mompeán et al. 2018; Pham et al. 2019).

Unlike other amyloid fibrils, commonly associated with neurodegenerative diseases, RHIM amyloids are functional and act as scaffolds for signal transduction (Li et al. 2012; Daskalov et al. 2021). A prominent example of their function is in necroptosis, a form of programmed cell death that depends on the activation of the pseudokinase MLKL. During canonical necroptosis, RIPK1 locally accumulates, facilitating the recruitment and heteromeric assembly with RIPK3 via RHIM-RHIM interactions (Li et al. 2012; Wu et al. 2014; Vanden Berghe et al. 2016; Mompeán et al. 2018). This heteromeric complex nucleates the formation of RIPK3 homopolymers, ultimately triggering MLKL oligomerization and membrane disruption (Samson et al. 2020; Clucas and Meier 2023). Additionally, RIPK1 can participate in other RHIM-based heteromeric complexes with ZBP1 (Z-DNA binding protein 1) and TRIF (TIR-domain-containing adapter-inducing interferon-β), further contributing to cellular defence mechanisms (Kaiser et al. 2008; Baker et al. 2022).

Despite their functional importance, the structural features of RIPK1 RHIM-based fibrils have remained elusive. In this assignment note, we report NMR backbone assignments on the RHIM-assembled amyloid of human RIPK1. These data provide a foundation for future studies aimed at understanding the molecular determinants of functional amyloid formation and how subtle sequence differences enable selective interactions within the RHIM signaling network.

## Methods and experiments

### Protein expression and purification

The human RIPK1 RHIM construct containing residues 496–583 was purchased from Genscript (New Jersey, NJ) with codons optimized for expression in *E. coli*. The construct was subcloned in a pET11a derived vector with an N-terminal His×6 tag. The plasmid was cloned and expressed in BL21 Star (DE3). For ^13^C and ^15^N uniformly labelled samples, protein was expressed following an adapted protocol described by Marley et al. (2001) and Sivashanmugam et al. (2009). Transformed cells were first cultivated at 37 °C in 2 L of LB medium until reaching an OD_600_ of 0.6–0.8, harvested by centrifugation, and finally resuspended and cultured at 25 °C in 0.5 L of M9 minimal medium supplemented with both ^13^C -D-glucose and ^15^NH_4_Cl ammonium chloride (Cambridge Isotope Laboratories) as the sole nitrogen and carbon sources. Protein overexpression was induced using 0.5 mM IPTG and left overnight to enhance isotope incorporation. For non-labelled samples, overexpression was induced directly in LB media using 0.5 mM IPTG and left overnight at 37 °C.

Once harvested, cells were resuspended in lysis buffer (50 mM Tris, 300 mM NaCl, and 1 µg/mL freshly prepared DNase) and lysed by sonication (30% amplitude, 5 s ON, 8 s OFF, for 10 effective minutes) on ice. Cell debris was removed by centrifugation at 30,000 rpm for 20 min at 4 °C, yielding an insoluble protein pellet (i.e. inclusion bodies). The inclusion bodies were resuspended in 1% SDS 150 mM NaCl, 50 mM Tris (pH 8.0) 1 mM DTT and sonicated (30% amplitude, 5 s ON, 8 s OFF, for 10 effective minutes) to enhance solubilization. Insolubilities were then removed from the sample by centrifugation at 30,000 rpm for 20 min at 4 °C. The His-tagged RIPK1 construct was loaded onto a pre-equilibrated HisTrap column (Cytiva). A 5-column-volume (5 CV) washing step was performed using the resuspension buffer (1% SDS, 150 mM NaCl, 50 mM Tris, pH 8.0). A second washing step was carried out with 0.5% SDS, 150 mM NaCl, and 50 mM Tris (pH 8.0). Elution was performed using the same buffer supplemented with 0.5 M imidazole (0.5 M imidazole, 0.5% SDS, 150 mM NaCl, 50 mM Tris, pH 8.0). The His-tag was retained and the protein was used without tag cleavage prior to fibril assembly.

### Fibril assembly and recovery

^13^C, ^15^N isotopically labelled and unlabelled proteins were mixed at a 1:4 ratio in 50 mM Tris-HCl (pH 7.4), 150 mM NaCl, 2% SDS to achieve the desired isotopic dilution while maintaining the proteins in non-assembled states. This material was diluted to 80 µM and dialyzed against Milli-Q water using a 3500 Da molecular weight cutoff dialysis membrane (Spectrum™ 123110) for 5 overnights at RT, with two water replacements. Fibrils were recovered by centrifugation cycles (1000 g, 15 min).

### NMR spectroscopy

Dialyzed fibrils from hRIPK1 were pelleted and transferred into a 3.2-mm rotor using home-made packing tools (Gelardo and Titaux-Delgado 2025) and a Ortoalresa Minicen RT255 centrifuge. Excess water was removed during the packing process. Approximately, 60 mg of wet fibrils were packed (corresponding to ~ 12.5 mg of isotopically labelled material). SSNMR experiments were conducted on a Bruker AVANCE NEO 600 MHz spectrometer (14.1 T) equipped with a HCN CPMAS cryogenically-cooled probe (Hassan et al. 2020). To establish sequential resonance assignments, three 3D spectra were recorded; namely, NCACX, NCOCX, and CANCOCX experiments, with 50 ms CORD mixing, which were corroborated and complemented using 2D ^13^C-^13^C CORD spectra with 5, 20 and 100 ms of mixing time (Pauli et al. 2001; Franks et al. 2007; Hou et al. 2013).

All experiments were collected at approximately 37 °C to maintain physiologically relevant conditions. Chemical shifts were indirectly referenced to DSS. Spectra were processed using Topspin 4.0 (Bruker Biospin) and analyzed with Sparky (D. Goddard and D. G. Kneller, SPARKY 3, University of California, San Francisco). Experimental details and acquisition parameters are provided in Table 1.

**Table 1:** Experimental NMR parameters

14.1 T (600 MHz 1 H frequency), 3.2 mm HCN CPMAS CryoProbe						
Experiments	2D NCA	2D NCACX	3D NCACX	3D NCOCX	3D CANCOCX	2D CORD
NS	8	8	8	8	64	4
MAS (kHz)	14	14	14	14	14	14
Sample T (K)	310	310	310	310	310	310
d1 (s)	2	2	2	2	2	2
Dec. power (kHz)	100	100	100	100	100	100
Transfer 1	HN-CP	HN-CP	HN-CP	HN-CP	HC-CP	HC-CP
CP time (ms)	1.00	1.00	1.00	1.00	1.20	1.75
Field Strength (kHz)	54(H);40(N)	54(H);40(N)	54(H);40(N)	54(H);40(N)	69(H);55(C)	69(H);55(C)
Transfer 2	NCA-CP	NCA-CP	NCA-CP	NCO-CP	CAN-CP	CORD
CP time (ms)	1.50	1.50	1.50	1.50	1.25	-
Field Strength (kHz)	4(N);18(C)	4(N);18(C)	4(N);18(C)	8.4(N);5.6(C)	3(C);11(N)	-
Mixing (ms)	-	-	-	-	-	5, 20, 100
Transfer 3		CORD	CORD	CORD	NCO-CP	
CP time (ms)		-	-	-	7.0	
Field Strength (kHz)		-	-	-	8.4(N);5.6(C)	
Mixing (ms)		50	50	50	-	
Transfer 4					CORD	
CP time (ms)					-	
Field Strength (kHz)					-	
Mixing (ms)					50	
F1	N	N	N	N	CA	C
Acq. time (ms)	11.0	7.9	7.9	7.9	6.0	17
Sweep width (kHz)	2.8	2.8	2.8	2.8	4.7	35
F2	CA		CA	CO	N	C
Acq. time (ms)	15.0	-	8.0	8.0	7.9	20
Sweep width (kHz)	52.6	3.5	3.5	3.5	2.8	44.2
F3		CX	CX	CX	CX	
Acq. time (ms)		15.0	15.0	15.0	17.4	
Sweep width (kHz)		52.6	52.6	52.6	58.8	

### Extended assignment

In order to access exclusively intra-residue information and facilitate protein assignment, 60 mg of an isotopically diluted sample containing a 25% of ^13^C, ^15^N - uniformly labelled RIPK1 (496–583), were packed into a 3.2 mm SiN rotor.

**Fig. 1:**
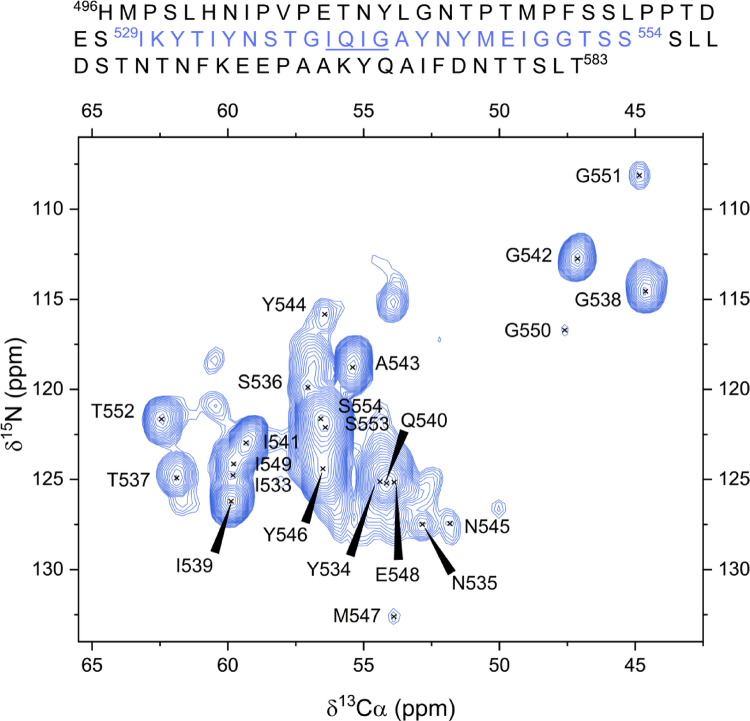
2D projection of CANCOCX spectrum of RIPK1 (496–583). Detected residues from the fibril core are annotated. The spectrum was collected on a 600 MHz spectrometer (^1^H frequency) at 298 K and MAS rate of 14 kHz

Only residues present in the rigid core of RIPK1 RHIM amyloid fibril were stiff enough to be detectable by cross-polarization (CP) transference experiments at ~ 37 °C (sample temperature). A sequential assignment of 26 residues (529–554) was achieved using a backbone walk strategy based on CA anchoring across 3D N_i_ and N_i+1_ based experiments. The 2D projection of the 3D ^15^N ^13^C CANCOCX spectrum (Fig. 1) displays signals corresponding to most of the assigned residues (533–554). Residues 529–532 exhibited low signal-to-noise ratios and could not be reliably distinguished in this projection. Additionally, due to the high ambiguity caused by the SSS repeat motif, residues S553 and S554 were excluded from further analysis.

The repetitive composition of the RIPK1 RHIM sequence—containing 5 isoleucines, 4 tyrosines, 4 glycines, 3 threonines and 3 serines within the 26 assigned residues (see Fig. 1) —posed a challenge for the unambiguous identification of certain side-chain resonances. Despite this, the resolution provided by 3D spectra enabled the complete (100%) unambiguous assignment of backbone heavy-atom nuclei (63 out of 63), and 93% of side-chain nuclei (54 out of 58) within residues 532–552. The only exceptions were the aromatic side chains, which remained partially ambiguous as a result of their highly similar chemical environments. For the three N-terminal residues (529–531), which did not yield signals in the NCA spectra, assignment was limited to the Cα atoms due to reduced signal intensity. Assignment was further supported by 2D ^13^C–^13^C CORD spectra acquired with short mixing times (5 and 20 ms), which revealed intra-residue correlations such as Cα–Cβ, Cα–CO, or Cβ–Cγ. In addition, spectra recorded with longer mixing times (100 ms) provided inter-residue cross-peaks, confirming the sequential connectivities established from 3D experiments.

**Fig. 2:**
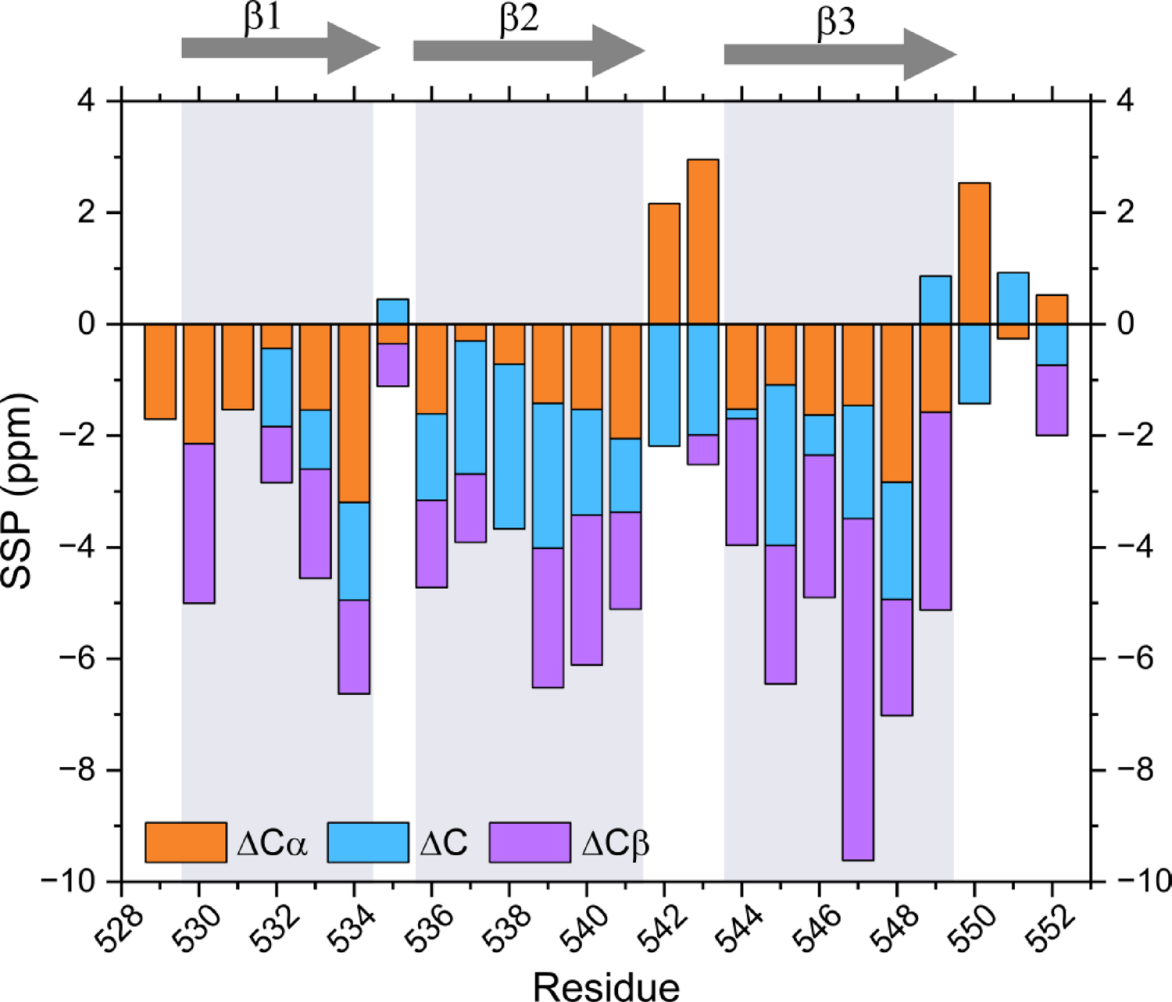
Secondary structure propensity (SSP) analysis of RIPK1 RHIM amyloid core (residues 529–552). Three regions compatible with β-strands are highlighted: 529–534, 536–541, and 544–549. ΔCα, ΔCO, and ΔCβ values represent the difference between experimental chemical shifts and random coil predictions. SSP values were calculated as ΔCα + ΔCO – ΔCβ; thus, ΔCβ contributions are inverted, making all bars point toward negative values in β-stranded regions, facilitating interpretation of secondary structure

The chemical shifts were compared with those predicted for a disordered conformation of the RIPK1 RHIM sequence using the Neighbour-Corrected Secondary Structure Propensity Calculator (Tamiola et al. 2010). Differences between experimental and predicted chemical shifts were used to derive secondary structure propensities (SSPs) for the different nuclei: ΔCA, ΔCO, ΔCB. A combined version of these indicators (ΔCA + ΔCO - ΔCB) is shown in Fig. 2. Negative values of ΔCA or ΔCO usually indicate the presence of β-sheets, while positive values suggest the formation of α-helices. In contrast, ΔCB behaves in the opposite way: positive values are associated with β-sheets, and negative values with α-helices. SSPs reveal that RIPK1 fibril core is formed by three clear β-strand regions, 529–534, 536–541 and 544–549. Following those stretches, residues 550–552 seem to be leading a floppy region with SSP values typical of disordered conformations.

In summary, using a cryogenically cooled CPMAS probe, the SSNMR characterization of human RIPK1 RHIM fibrils was accomplished on an isotopically diluted sample. A total of 26 assigned residues adopt extended conformations organized in three β-strands, as gauged from the SSP analysis. These NMR chemical shifts constitute a solid basis for structural and dynamic studies of RIPK1 and related RHIM-containing assemblies, and are deposited in the Biological Magnetic Resonance Data Bank (BMRB) under accession number 34,971.

## Data Availability

Assignments for the RIPK1 RHIM fibrils have been deposited in the BMRB (https://bmrb.io) under accession 34971. The plasmid used in this study is available upon request.
